# Viscerotropic disease and acute uveitis following yellow fever vaccination: a case report

**DOI:** 10.1186/s12879-020-4838-x

**Published:** 2020-02-10

**Authors:** Lev Volkov, Gilda Grard, Pierre-Edouard Bollaert, Guillaume A. Durand, Aurélie Cravoisy, Marie Conrad, Lionel Nace, Guilhem Courte, Rémy Marnai, Isabelle Leparc-Goffart, Sébastien Gibot

**Affiliations:** 1 CHRU de Nancy, Intensive Care Unit, Réanimation Médicale Hôpital Central-CHU de Nancy, 29 avenue du maréchal de Lattre de Tassigny, 54035 Nancy, France; 2 Institut de Recherche Biomédicale des Armées, National Reference Laboratory for arboviruses, Marseille, France; 3 Unité des Virus Emergents (UVE: Aix-Marseille Univ - IRD 190 - Inserm 1207 - IHU Méditerranée Infection), Marseille, France

**Keywords:** Yellow fever vaccine, Yellow fever vaccine-associated viscerotropic disease, Uveitis

## Abstract

**Background:**

Yellow fever vaccine exists for over 80 years and is considered to be relatively safe. However, in rare cases it can produce serious neurotropic and viscerotropic complications. We report a case of a patient who presented both viscerotropic and neurological manifestations after yellow fever vaccination.

**Case presentation:**

We describe the case of a 37 years old man who developed after the yellow fever vaccination a yellow fever vaccine-associated viscerotropic disease followed by acute uveitis. Prolonged detection of yellow fever RNA in blood and urine was consistent with yellow fever vaccine-associated adverse event. The final outcome was good, although with persistent fatigue over a few months.

**Conclusions:**

Even if the yellow fever vaccine is relatively safe, physicians should be aware of its possible serious adverse effects.

## Background

Yellow fever is an acute hemorrhagic disease caused by the yellow fever virus (YFV), a ribonucleic acid virus member of the genus Flavivirus. It is transmitted to humans by infected mosquitos of the genus *Aedes* and *Haemogogus* that acquire the virus by feeding on infected human or nonhuman primates [1]. Yellow fever is endemic in tropical areas of Africa and Central and South America, with occasional epidemic outbreaks. It causes fever with headache, myalgias, arthralgias, vomiting, hepatitis with jaundice and can be responsible for renal failure and hemorrhagic syndrome. Severe yellow fever can be fatal in 20–60% of all cases [2]. There is no specific antiviral treatment available. Yellow fever vaccine exists for over 80 years [3] and has been successfully used to control the disease in many endemic countries. A single dose provides a long term immunization in nearly all vaccinated individuals [1, 4]. The vaccine currently used in Europe contains the live attenuated yellow fever 17D-204 substrain derived from the wild type Asibi strain. The 17D virus has a restricted replication and attenuated neurotropism and viscerotropism as compared to the wild-type virus [4]. Vaccination can sometimes cause mild adverse effects such as myalgia, headache, and slight fever associated with low, transient viremia. However, serious neurotropic and viscerotropic complications can occur in rare cases.

## Case presentation

We report the case of a 37 years old man, previously healthy, with no medical history, no treatment, and no travels abroad. As a child, he presented repeated morbilliform skin eruptions with one episode diagnosed as measles. In preparation for work-related travel to Mali, he was vaccinated with yellow fever 17D-204 vaccine (Sanofi Pasteur STAMARIL n°P3M361V) and hepatitis A vaccine (MSD Vaccins VAQTA n°R020782) in the left arm, and with meningitis A, C, Y, W135 vaccine (Pfizer NIMENRIX n°W78068) in the right arm, all in 1 day. He received the yellow fever vaccine for the first time. After 4 days, he developed a fever between 38° and 40 °C with chills. Three days later a non-productive cough appeared, together with dyspnea, malaise, sore throat, and non-bloody diarrhea, followed by a morbilliform skin rash of the chest. He consulted at the emergency department of Nancy University Hospital on the eighth day after the vaccine, in November 2018.

Blood pressure was 106/73 mmHg, oxygen saturation rate was 99%, pulse rate was 103 beats/minute and respiratory rate was 25/min. The temperature was 39.3 °C.

**Fig. 1:**
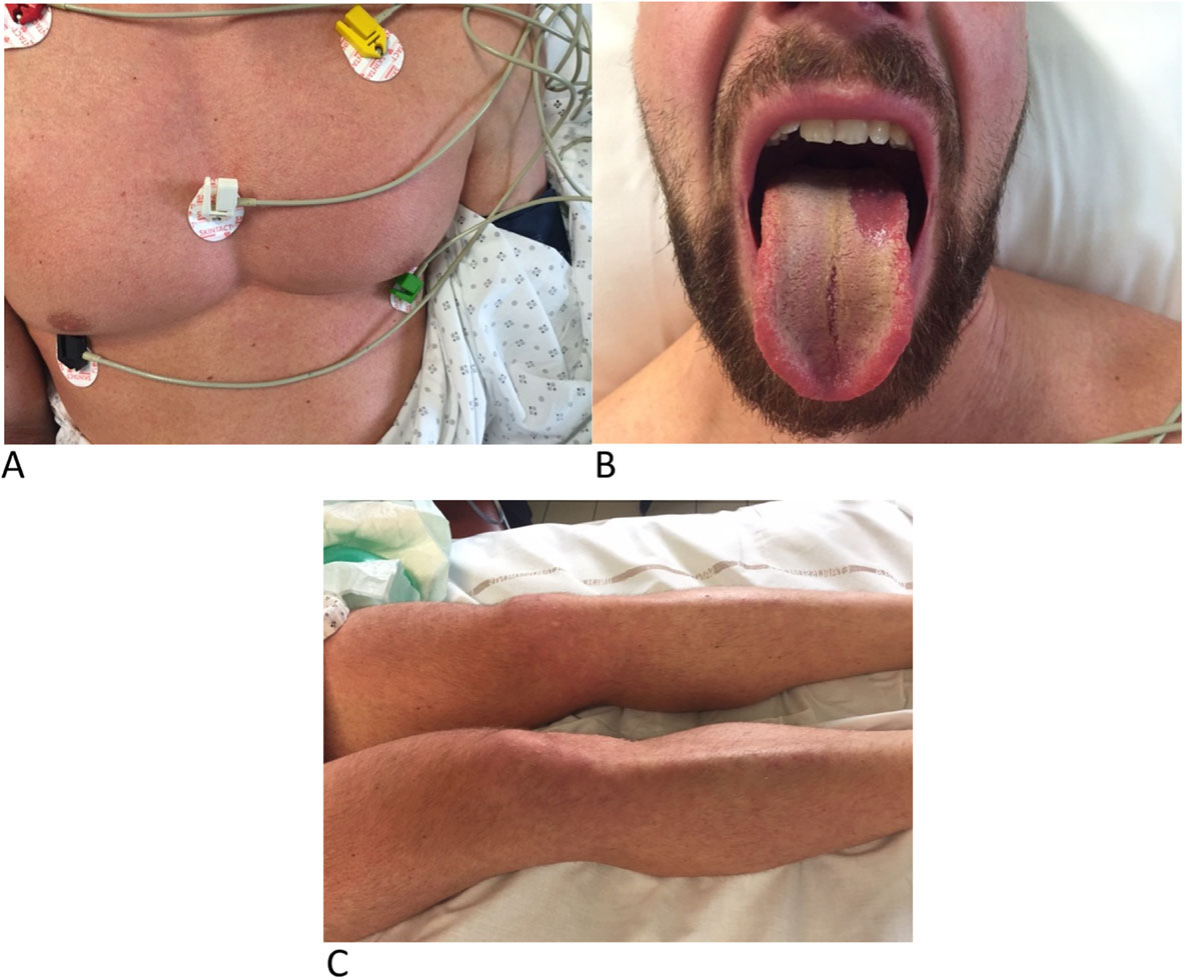
Cutaneo-mucous manifestations. **a** Thoraco-abdominal skin rash. **b** Strawberry tongue. **c** Lower limbs skin rash

On examination, he had a thoraco-abdominal skin rash (Fig. 1) without purpura, red conjunctiva, pharyngitis, right hypochondria pain, and a strawberry tongue (Fig. 1). Laboratory results showed thrombocytopenia of 46 G/L (normal range is 150–450 G/L), lymphopenia of 0.41 G/L (normal range is 1–4 G/L), with a total blood cell count of 4.62 G/L (normal range is 4–10 G/L). Hemoglobin was 15.5 g/dl (normal range is 13–17 g/dL). Liver enzymes were elevated with aspartate aminotransferase 428 UI/L (normal range is 13–40 U/L) and alanine aminotransferase 309 UI/L (normal range is 7–40 U/L). Total bilirubin concentration was raised to 42 micromol/L (normal range is 5–21 micromol/L). Renal function was normal: creatinine 99 micromol/L (normal range is 64–104 micromol/L) with hyponatremia of 131 mmol/L (normal range is 136–146 mmol/L). Serum CRP level was raised to 179.9 mg/L (normal range is< 5 mg/L) and serum lactate level was raised to 2.1 mmol/L (normal range is< 1.6 mmol/L). The chest radiography was normal. The patient was admitted to the ICU.

In ICU, examination showed hepatomegaly, and inguinal and axillary adenopathies. The skin rash had extended to the lower limbs (Fig. 1). The patient developed an occipital headache. Laboratory results worsened with aspartate aminotransferase concentration raised to 621 UI/L, alanine aminotransferase 506 UI/L, total bilirubin 54 micromol/L, and lymphopenia lowered to 0.11 G/L. White blood cell count remained in the normal range (4–10 G/L), hemoglobin was 15 g/dL, prothrombin time was 91% (normal range is 70–100%) and renal function remained normal (creatinine< 104 micromol/L). The patient was empirically treated with Ceftriaxone and Spiramycin. His condition progressively improved, the skin rash disappeared, platelets count increased, whereas liver enzymes decreased. He was released after 6 days of observation, on the 15th day after vaccination. Fever persisted for another four days (38.5 °C).

A few days after the discharge he had a haze in the right eye, without any other symptoms, and consulted the ophthalmology emergency department. His visual acuity was not reduced (10/10 in both eyes). The conclusion of the ophthalmologic examination was an acute anterior and intermediate hypertensive uveitis with neither papillitis nor vasculitis. Physical examination was normal, adenopathies had disappeared. He was treated with topical atropine, beta-blockers, corticosteroids and subconjunctival injections of corticosteroids. As a result, he fully recovered within one month, although fatigue persisted over a few months.

Serological studies were negative for HIV, syphilis, hepatitis A, hepatitis C, hepatitis E, Ebstein-Barr virus and Lyme, and showed protective post-vaccinal immunity for hepatitis B. Measles serology was positive for IgG and negative for IgM. Cytomegalovirus (CMV) serology was positive for IgM and IgG, but blood polymerase chain reaction (PCR) didn’t show any CMV viremia. Blood and urine cultures, as well as urinary antigens for *Legionella pneumophila*, were negative. Serum protein electrophoresis showed polyclonal hypergammaglobulinemia. PCR for dengue, West-Nile virus, tick-borne encephalitis (TBE) and Chikungunya were negative.

**Fig. 2:**
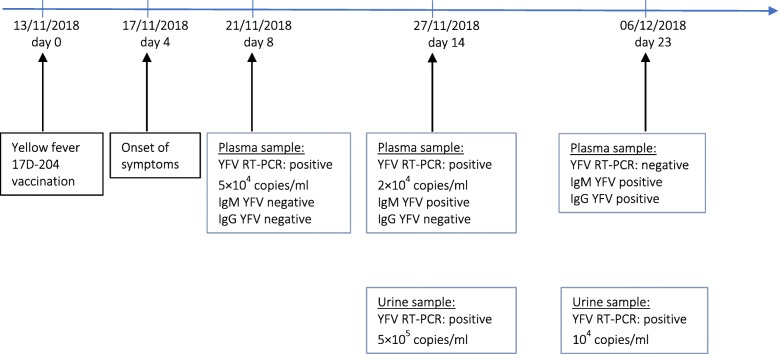
Biological timelines for YFV RNA detection and YFV antibodies response in plasma and urine samples

YFV RNA was detected in plasma samples on the 8-th and 14-th post-vaccination days, with the highest viral load of 5 × 10^4^ RNA copies/ml (5 × 10^2^ TCID50/ml) on the 8-th day. YFV RNA was detected in urine samples on the 14-th and 23-rd days with the highest viral load of 5 × 10^5^ RNA copies/ml (5 × 10^3^ TCID50/ml) on the 14-th day (Fig. 2). Viral load was quantified by RT-PCR [5] using a range of quantified RNA transcripts and a range of titrated viruses (in TCID50/ml). Viral titration in cell culture was not performed. The presence of YFV RNA in the plasma until the 14-th and in urine until the 23-rd post-vaccination days was consistent with a yellow fever vaccine associated adverse event.

## Discussion and conclusions

Since the 1930s many studies have shown that the yellow fever vaccine is relatively safe, with more than 90% of reported adverse events not being serious [6]. It can cause mild adverse effects during the first week after administration, such as myalgia, headache, and asthenia in 30% of cases, as well as slight fever [4].

Neurologic adverse effects associated with yellow fever vaccine are occasionally reported since the 1950s. In very rare cases, yellow fever vaccination can cause encephalitis and is possibly associated with Guillain-Barre Syndrome and acute disseminated encephalomyelitis (ADEM) [7].

Viscerotropic complications following the vaccine are termed “yellow fever vaccine-associated viscerotropic disease (YEL-AVD)”. YEL-AVD typically appears within one week after vaccination. Symptoms are similar to the wild yellow fever virus: fever, headache, myalgia, vomiting, and diarrhea. Then can occur thrombocytopenia, liver enzymes elevation, jaundice, renal dysfunction, and in severe cases acute multiple organ failure [1, 8]. YEL-AVD is fatal in 65% of reported cases [1, 8]. The first known description of a suspected viscerotropic adverse effect was made in 1973 [9]. YEL-AVD is estimated to occur at a frequency of 0.3–0.4 per 100,000 yellow fever doses distributed [8]. It is suspected to be caused by dissemination and replication of the life attenuated vaccine virus. YEL-AVD has been reported with different virus substrains, and no remarkable genetic variation has been found in most cases [10, 11]. In our case, the detected virus was not sequenced. Factors favorizing YEL-AVD occurrence are probably host-related. The only identified risk factors are the age of more than 60 years and history of thymus disease or thymectomy, although interference with the immune response seems to be contributing as well [11]. An auto-immune disease might be a risk factor for YEL-AVD [1], even though several case reports of severe adverse events associated with YFV vaccine describe patients without any known immunocompromising medical history [12–15].

Our case is consistent with a YEL-AVD as defined by the onset of symptoms within the week following yellow fever vaccination with fever, dyspnea, malaise, abnormal laboratory findings with thrombocytopenia, elevation of liver enzymes and total bilirubin, with no evidence of other diagnoses [1, 8]. Following the Brighton Collaboration case definition of viscerotropic disease [8], our case has a level 2 of diagnostic certainty with the presence of hepatic failure (total bilirubin is ≥1.5 of the upper limit and liver enzymes are ≥3 of the upper limit), platelet disorder (platelets are < 100 G/L) and tachypnea (> 20/min). One of the differential diagnoses could have been scarlet fever, but the skin rash was different from the typical scarlatiniform rash, without desquamation, and pharyngitis didn’t involve the tonsils. Even if no laboratory tests for scarlet fever were performed, the clinical and biological presentation was not consistent with this diagnosis. Skin rash is not typically associated with YEL-AVD, but a transient erythematous rash of the trunk and limbs has been described [13]. Other exanthematous diseases were eliminated by laboratory tests in our patient (measles, EBV, CMV, HIV, syphilis and other arboviruses). Since no histological analyses were performed, laboratory confirmation of YEL-AVD was made by the prolonged presence of YFV RNA in plasma and urine after 7 days that followed the vaccination**.** Indeed, a possible urine excretion of YFV RNA has been shown during the first 7 days following vaccination, but persistent urine excretion after 7 days was found in patients with suspected yellow fever vaccine-associated adverse events [16].

Acute anterior and intermediate uveitis presented by our patient 2 to 3 weeks after YFV vaccination could be related to the vaccine. Yellow fever vaccination is known to be associated with uveitis and other ophthalmologic manifestations such as vasculopathy and optic neuritis, even if the frequency of these manifestations is unknown [17]. The cerebro-spinal liquid analysis was not performed in our case. Furthermore, uveitis could be a manifestation of an unknown underlying immunological disease.

Viscerotropic and neurologic adverse effects following YFV vaccine are rare but well-described complications with potentially fatal outcome. Our patient presented yellow fever vaccine-associated viscerotropic disease followed by uveitis. The final outcome was good, although with persistent fatigue for several months. Even if YFV vaccine has been used for years and has shown to be very efficient for epidemical control of yellow fever, physicians should be aware of the potential serious adverse effects and seek the presence of risk factors before the vaccination.

## Data Availability

The datasets used and/or analyzed during the current study are available from the corresponding author on reasonable request.

## Abbreviations

CMV: Cytomegalovirus
CRP: C-reactive protein
HIV: Human immunodeficiency virus
ICU: Intensive care unit
Ig: Immunoglobulin
PCR: Polymerase chain reaction
RT-PCR: Reverse transcription polymerase chain reaction
TBE: Tick-borne encephalitis
TCID50: Median tissue culture infectious dose
YEL-AVD: Yellow fever vaccine-associated viscerotropic disease
YFV: Yellow fever virus
